# Design of *N*-Way Power Divider Based on TE_10_ Mode Splitting Strategy

**DOI:** 10.3390/mi16091033

**Published:** 2025-09-10

**Authors:** Jianfeng Chen, Haidi Tang, Shengqi Zhang, Leijun Xu

**Affiliations:** School of Electrical and Information Engineering, Jiangsu University, Zhenjiang 212013, China; tang20020322@163.com (H.T.); nanfu0011@163.com (S.Z.)

**Keywords:** TE10 mode, mode converter, power divider, waveguide transitions

## Abstract

This paper presents a novel 1-to-*N* power division architecture combining overmoded ${\mathrm{TE}}_{10}$ mode waveguides and modular *N*-way waveguide-to-microstrip mode converters. By decomposing the ${\mathrm{TE}}_{10}$ mode field distribution along the narrow wall of a rectangular waveguide, the proposed design enables flexible power splitting into arbitrary output ports (even or odd numbers) through uniform sub-${\mathrm{TE}}_{10}$-mode waveguide pathways. To achieve the above function using microwave transmission lines, a tapered transition structure ensures wideband excitation of the overmoded waveguide, while linearly tapered slot antennas (LTSAs) serve as *N*-way mode converters. Prototypes with two-, three-, and four-channel outputs demonstrate excellent amplitude-phase uniformity (≤0.5 dB amplitude imbalance and ≤$5^{\circ }$ phase deviation) across 6.5–12 GHz, with return loss <−10 dB. The modular 1-to-*N* power divider enables the rapid reconfiguration of output channels by simply replacing the mode converter module.

## 1. Introduction

**Figure 1:**
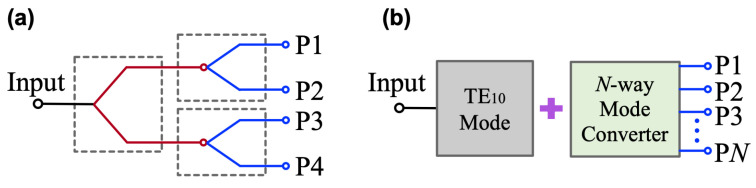
(**a**) Traditional design with cascaded T-junctions to increase output ports. (**b**) Proposed power division scheme.

Power combination and division techniques play a critical role in modern microwave systems. By splitting power flow into multiple branches, these techniques enable key functionalities including array antenna excitation (feeding networks) [1] and distributed power amplification [2,3]. Conventional power divider architectures, such as Wilkinson and Gysel dividers [4,5], are particularly suitable for binary power division designs. For configurations requiring more output ports, tree-type or chain-type structures [6,7] can be implemented through cascaded T-junctions to exponentially increase output ports, at the expense of significantly increased physical dimensions, as depicted in Figure 1a. Moreover, these conventional approaches face inherent limitations in achieving odd-numbered power division ratios, which imposes practical constraints on their applications, and distributed power amplification [2,3]. Conventional power divider architectures, such as Wilkinson and Gysel dividers [4,5], are particularly suitable for binary power division designs. For configurations requiring more output ports, tree-type or chain-type structures [6,7] can be implemented through cascaded T-junctions to exponentially increase output ports at the expense of significantly increased physical dimensions, as depicted in Figure 1a. Moreover, these conventional approaches face inherent limitations in achieving odd-numbered power division ratios, which imposes practical constraints on their applications.

Significant efforts have been devoted to addressing these challenges. Planar transmission technologies like [8,9] have been developed to realize nonbinary power division. Additionally, radial power division strategies offer flexible spatial/3D power distribution capabilities, where rotational symmetry in circular distribution networks ensures uniform amplitude-phase characteristics across all output ports [10,11,12,13]. In [14,15], high-order ${\mathrm{TE}}_{n0}$ modes in rectangular waveguides are exploited for power division. Since ${\mathrm{TE}}_{n0}$ modes can be decomposed into *N* individual ${\mathrm{TE}}_{10}$ mode components, they naturally serve as *N*-way power distribution networks, motivating extensive research on high-purity ${\mathrm{TE}}_{n0}$ mode excitation [16,17,18,19]. Recently, a multiport power divider utilizing epsilon-near-zero (ENZ) metamaterials was proposed [20]. This design enables equal-amplitude and in-phase power distribution across outputs independent of geometric symmetry, with arbitrary power division ratios achievable through output waveguide width adjustment. However, the narrow operational bandwidth associated with ENZ effects remains a critical limitation.

In this paper, a novel modular 1-to-*N* power division architecture combining an overmoded ${\mathrm{TE}}_{10}$ mode waveguide with an *N*-way waveguide-to-microstrip mode converter is presented. The ${\mathrm{TE}}_{10}$ mode in rectangular waveguides maintains its propagation characteristics determined by the wide-side dimension [21,22,23], enabling decomposition into *N* identical sub-${\mathrm{TE}}_{10}$ mode waveguides along the narrow-side direction. Multiple identical linearly tapered slot antennas (LTSAs) are integrated to convert each sub-${\mathrm{TE}}_{10}$ mode to microstrip signals. Figure 1b illustrates the proposed power division scheme. Compared with conventional 1-to-*N* power dividers requiring complete system redesign for different output configurations, this approach only necessitates replacement of the *N*-way mode converter while retaining the input waveguide structure. To validate the methodology, prototypes with two-, three-, and four-channel outputs were implemented using different *N*-way mode converters. Both simulated and measured results demonstrate good agreement, confirming the feasibility of the proposed design paradigm.

## 2. Methods

**Figure 2:**
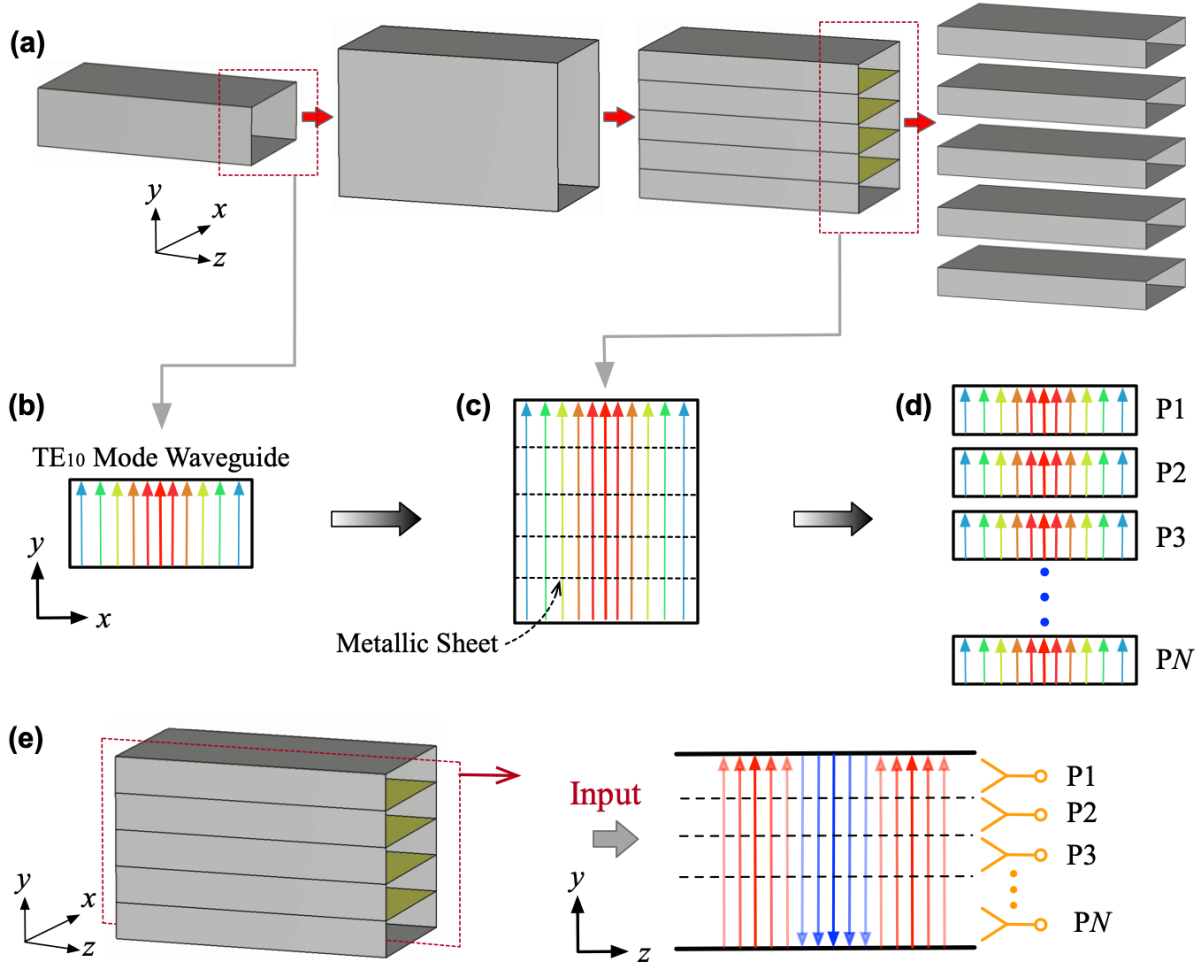
(**a**) Evolution from traditional rectangular waveguide to *N*-way output channels. (**b**) Transverse comparison of ${\mathrm{TE}}_{10}$ mode characteristics in rectangular waveguides with varying narrow wall heights, where (**c**) several metallic sheets are loaded inside the waveguide and (**d**) subdivide the structure into *N* individual ${\mathrm{TE}}_{10}$ mode waveguides. (**e**) Longitudinal cross-sectional illustration of ${\mathrm{TE}}_{10}$ mode propagation terminated by an *N*-way mode conversion interface.

In general, electromagnetic (EM) waves propagating in rectangular waveguides must operate in specific modes to achieve efficient transmission, such as the ${\mathrm{TE}}_{mn}$ mode. Here, *m* and *n* are mode indices corresponding to the field distributions along the *x*- and *y*-axes. When either index equals zero, it indicates a uniform field distribution in that direction, and the cutoff frequency and dispersion characteristics are solely determined by the dimension in the other direction of the waveguide. For instance, the ${\mathrm{TE}}_{10}$ mode shown in Figure 2a,b has its cutoff frequency $f_{c}$ and propagation constant β expressed as:

(1) $$f_{c}=\frac{c}{2a}$$

(2) $$\beta =\frac{2\pi }{c}\sqrt{f^{2}-{f_{c}}^{2}}$$

Here, we define the width and height of the waveguide as *a* and *b*, respectively. According to (1) and (2), increasing the waveguide height *b* along the *y*-axis preserves the mode characteristics, as illustrated in Figure 2b.

Ideally, conducting boundary conditions require electric fields to be perpendicular to the surface. For the ${\mathrm{TE}}_{10}$ mode in Figure 2, the electric field remains perpendicular to the *x*-axis. In this scenario, inserting a metallic sheet parallel to the *x*-axis within the waveguide does not perturb the original field distribution, as indicated by the dashed lines in Figure 2c. Additionally, the waveguide structure in Figure 2b can be subdivided along the metallic sheet into *N* individual ${\mathrm{TE}}_{10}$ mode waveguides with identical propagation characteristics. This enables a power division process from one input to *N* outputs, transitioning from Figure 2b–d.

**Figure 3:**
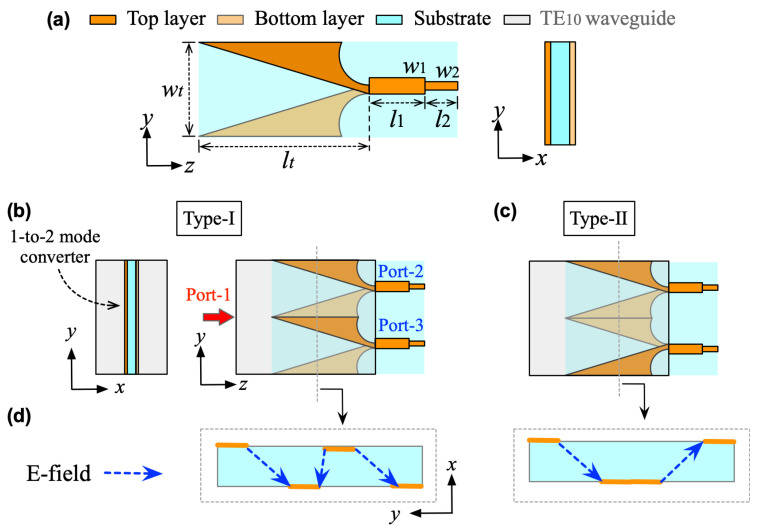
(**a**) Design details of the fundamental element in the proposed *N*-way mode converter with different combination schemes. (**b**) Type-I with translational symmetry. (**c**) Type-II with mirror symmetry. (**d**) E-fields in the transverse section of the mode converters. ($w_{1}$ = 2.9, $w_{2}$ = 2, $w_{t}$ = 18, $l_{1}$ = 11, $l_{2}$ = 9, $l_{t}$ = 35. Unit: mm).

In practice, the metallic sheet in Figure 2c is not strictly required for physical implementation since it introduces no mode perturbation. A 1-to-*N* power division can alternatively be achieved by incorporating several waveguide-to-microstrip transitions with identical transmission characteristics, as illustrated in Figure 2e. Figure 3 presents the design details of the two-way mode converter. Here, a LTSA is employed to realize the conversion from the ${\mathrm{TE}}_{10}$ mode with *y*-polarization to the microstrip line. By combining multiple LTSAs and inserting them into the rectangular waveguide at the center along the *x*-axis edge, a 1-to-2 power division is achieved.

**Figure 4:**
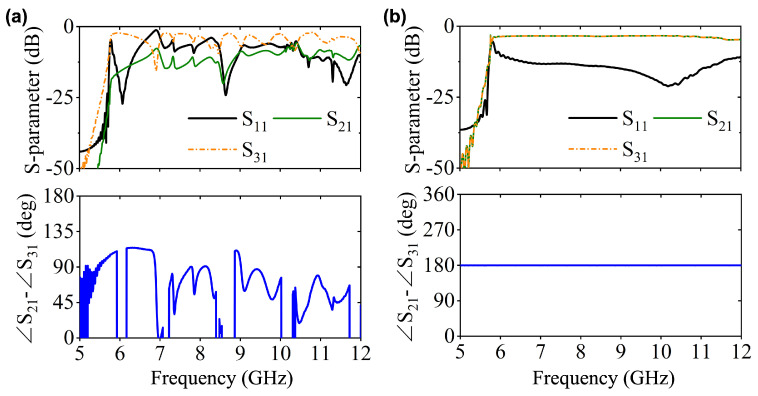
Simulated performance of (**a**) Type-I and (**b**) Type-II structures, including S-parameters and phase differences between adjacent ports.

**Figure 5:**
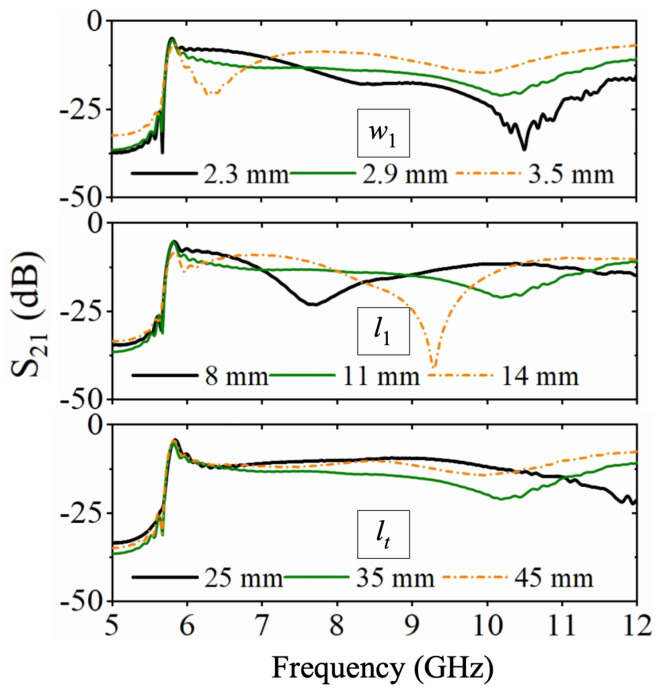
Simulation of $S_{21}$ results for different parameter based on Type-II structure.

Notably, there are two configurations to assemble the LTSAs for implementing two-way mode conversion, as shown in Figure 3b,c. For the Type-I structure, the two LTSA pairs exhibit translational symmetry, while the Type-II configuration features mirror symmetry between the LTSA pairs. Figure 4 compares the simulated performance of both, including S-parameters and phase differences between adjacent ports. For Type-I, significant fluctuations in transmission characteristics and phase differences are observed within the operating band. This is primarily attributed to the translational symmetry of Type-I, where the adjacent regions of the two LTSAs lack continuity. This discontinuity prevents the EM waves from converting into the microstrip mode along the slot direction of the LTSA. This result is further demonstrated by the E-field distribution plot in the left panel of Figure 3d. In contrast, the Type-II structure demonstrates superior transmission performance, showcasing the inherent broadband capability of the LTSA architecture. Benefiting from mirror symmetry, the output ports maintain equal amplitude with a stable $180^{\circ }$ phase difference, which makes it suitable for out-of-phase power divider designs. Figure 5 presents the transmission characteristics of a 1-to-2 power divider based on the Type-II structure under different structural parameters. Here, the $l_{1}$ and $w_{1}$ parameters of the microstrip line section are primarily designed to achieve impedance matching between the LTSA and the 50 Ω microstrip line, thereby reducing the return loss. The LTSA achieves a wider operational bandwidth when its length lt is set to 35 mm.

For ${\mathrm{TE}}_{10}$ mode waveguides, employing a Type-II configuration enables highly flexible design of power dividers with *N*-way output ports. However, increasing the number *N* enlarges the mode converter’s total width ($N\times w_{t}$). As Figure 3 shows, the rectangular waveguide height *b* equals this width. When *N* increases further, *b* eventually exceeds the waveguide width a (Figure 2a), causing the ${\mathrm{TE}}_{10}$ mode to propagate in an overmoded waveguide. Consequently, another critical challenge arises regarding the efficient excitation of ${\mathrm{TE}}_{10}$ mode in overmoded waveguides.

**Figure 6:**
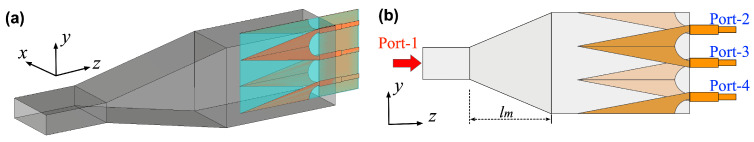
Configuration of the proposed 1-to-3 power divider with tapered transition structure between standard waveguide and overmoded waveguide. (**a**) Three-dimensional view. (**b**) Longitudinal cross-section view.

A traditional, coaxial probe can be employed to excite a rectangular waveguide. However, in overmoded waveguides, direct application of this method may excite higher-order modes, degrading the power divider’s performance. To address this, we implement a tapered waveguide in this work to connect the standard waveguide with the over-moded waveguide. This structure enables a gradual transition of the pure ${\mathrm{TE}}_{10}$ mode into the overmoded waveguide, optimizing both transmission bandwidth and efficiency, as shown in Figure 6. Here, a power divider with odd-numbered output ports (3 ports) is designed as an example to demonstrate the feasibility of the proposed design paradigm.

**Figure 7:**
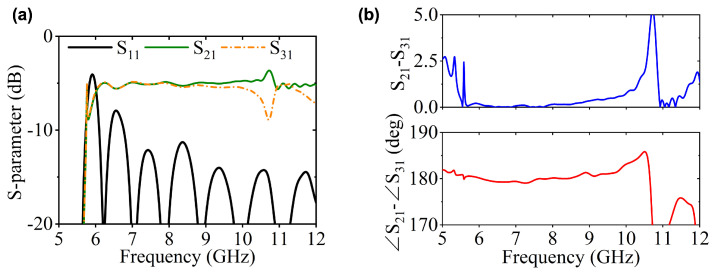
Transmission performance in tapered structure. (**a**) S-parameters. (**b**) Amplitude and phase differences between adjacent output ports.

Based on above design principle, a power divider with odd-numbered output ports (3 ports) is designed and presented in Figure 6 as an example to demonstrate the feasibility of the proposed design paradigm. The variation in return loss is given in Figure 7a. Notably, the ${\mathrm{TE}}_{10}$ mode maintains good output performance ($S_{11}$ < −10 dB) across 6.5–12 GHz. The amplitude and phase imbalance between adjacent ports are shown in Figure 7b. At lower frequencies, the amplitude and phase imbalances remain minimal. However, as the frequency increases, the amplitude imbalance gradually intensifies, reaching an extremum near 10.7 GHz. Correspondingly, significant phase fluctuations emerge above 10.7 GHz.

**Figure 8:**
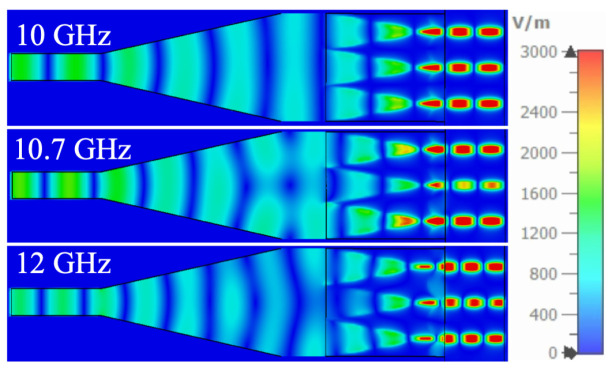
Snapshots of the electric field in the proposed 1-to-3 power divider.

To investigate the amplitude-phase imbalance mechanism at high frequencies, instantaneous E-field distributions at distinct frequencies are analyzed in Figure 8. At 10 GHz, the field distributions across the three output ports exhibit high consistency. However, at 10.7 GHz, field splitting occurs during propagation. By 12 GHz, the propagating field undergoes a transition from planar to spherical wavefronts along the taper section. This results in nonuniform E-field distribution along the *y*-axis when EM waves propagate into the overmoded waveguide, which further accounts for the high-frequency amplitude and phase fluctuations observed in Figure 7b.

**Figure 9:**
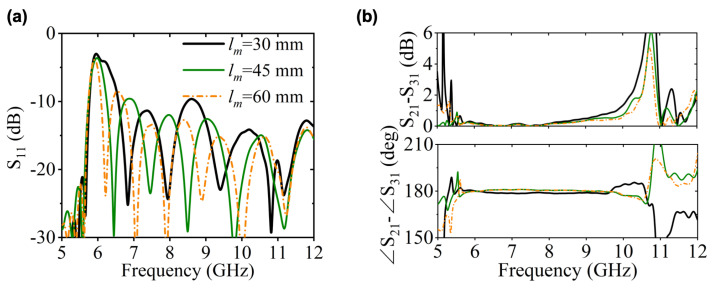
Transmission performance of different tapered transition structure length $l_{m}$. (**a**) S-parameters. (**b**) Amplitude and phase differences between adjacent output ports.

It should be emphasized that our tapered structure can be conceptually analogous to a rectangular horn antenna. According to antenna theory, when the *E*-plane radiation aperture exceeds specific wavelengths, phase discrepancies between edge and center regions cause main lobe splitting in radiation patterns [24]. Similarly, in our design, excessive height (*y*-axis direction) in the overmoded waveguide inevitably leads to significant amplitude reduction at the central output port due to inherent field distribution characteristics, as observed at 10.7 GHz in Figure 7b. Figure 9 presents the transmission performance under various tapered transition lengths $l_{m}$. It can be observed that when $l_{m}$ is small, the amplitude and phase consistency at high frequencies deteriorates significantly. When lm is larger, the fields within the two rectangular waveguides of different dimensions transition more smoothly.

## 3. Results

**Figure 10:**
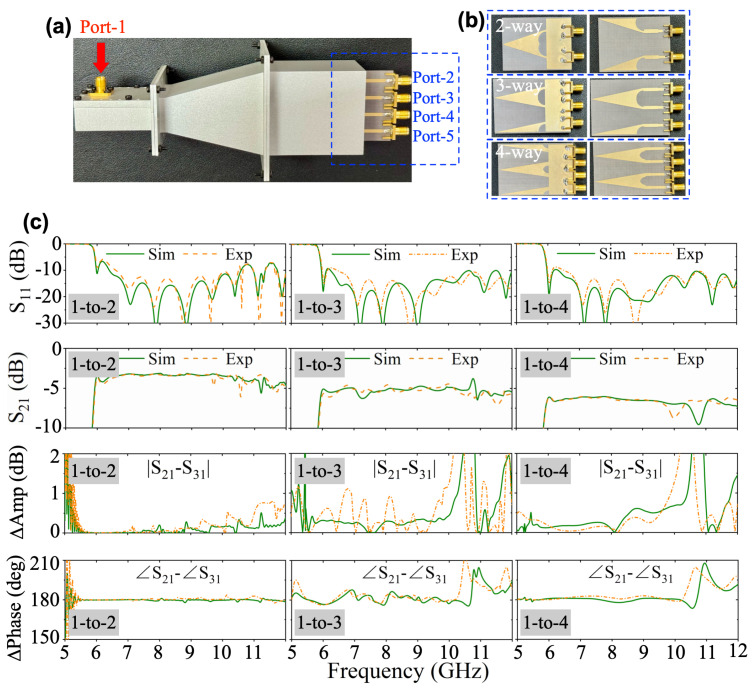
Fabricated prototype of the proposed (**a**) power divider and (**b**) two-, three-, and four-way mode converters. (**c**) Simulated and measured results.

To validate the proposed design methodology, a rectangular waveguide power divider incorporating three distinct output modules (two-, three-, and four-way mode converters) is fabricated and characterized, as illustrated in Figure 10. As discussed above, reconfigurable port-count operation is achieved by replacing the output modules without modifying the waveguide structure. The simulated and measured S-parameters are comparatively analyzed in Figure 10, demonstrating consistent performance across configurations. Notably, $S_{11}$ maintains values below −10 dB over most of the operating frequency (6–12 GHz), irrespective of module type. Figure 10c presents amplitude and phase-balancing characteristics between adjacent output ports. For the 1-to-2 power division case, structural symmetry ensures better performance in amplitude and phase balance. In addition, the 1-to-3 and 1-to-4 configurations present distinctive behaviors. The amplitude imbalance exhibits a resonance peak near 10.7 GHz while phase distortion progressively deteriorates at higher frequencies. These observations align with the field distribution analyzes presented in Figure 7 and Figure 8.

## 4. Conclusions

In conclusion, we propose a novel 1-to-*N* power division architecture based on overmoded ${\mathrm{TE}}_{10}$ mode waveguides and *N*-way mode converter. By exploiting the field decomposition characteristics of ${\mathrm{TE}}_{10}$ modes in rectangular waveguides, the design enables flexible power splitting into arbitrary output ports (even or odd numbers) through modularized waveguide-to-microstrip transitions. A tapered structure is implemented to achieve wideband, high-efficiency conversion of ${\mathrm{TE}}_{10}$ modes from standard to overmoded waveguides; LTSAs serve as the *N*-way mode converter for multiport power distribution. The proposed architecture decouples port configuration from input waveguide design, allowing facile adjustment of output channel counts by replacing only the *N*-way mode converter.

## Data Availability

The data that support the findings of this study are available from the corresponding authors upon reasonable request.
